# What’s in it for me? A mixed-methods study on teachers’ value creation in an inter-institutional community on open educational resources in higher education

**DOI:** 10.1007/s10639-022-11424-7

**Published:** 2022-11-08

**Authors:** Marjon Baas, Robert Schuwer, Ellen van den Berg, Tjark Huizinga, Roeland van der Rijst, Wilfried Admiraal

**Affiliations:** 1 ICLON-Graduate School of Teaching, Leiden University, Kolffpad 1, 2333 BN Leiden, The Netherlands; 2 School of Education, Saxion University of Applied Sciences, Van Galenstraat 20, 7511 JL Enschede, The Netherlands; 3 School of ICT, Fontys University of Applied Sciences, Rachelsmolen 1, 5612 MA Eindhoven, The Netherlands; 4 Centre for the Study of Professions, Oslo Metropolitan University, Pilestredet 40, N-0170 Oslo, Norway

**Keywords:** OER, Open Educational Resources, Communities, Value Creation Framework, Teachers, Higher Education

## Abstract

The affordances of Open Educational Resources (OER) have resulted in various initiatives around the world, but most of them cease to exist once the initial project funding stops. Communities might be a means to create sustainable practices, yet, such communities can only function if their members perceive these communities as valuable. We applied the value creation framework of Wenger, Trayner, and De Laat to examine the value teachers ascribe to their engagement with an inter-institutional community on OER. In this community, 15 universities of applied sciences collaborated on sharing knowledge and resources across their institutional barriers. We collected data through user statistics, an online questionnaire, and semi-structured interviews. Major value creation occurred from teachers’ personal needs, with dominant immediate and potential values. Findings on applied and realized values denote that it became easier for teachers to connect with peers, and to initiate collaboration projects across institutes. The framework we used is helpful to inform actions to further promote value creation in communities on OER. Recommendations relating to communities’ aspirations, its relations with the wider organization, and adoption of OER are formulated to inform sustainable practices of inter-institutional communities.

## Introduction

Teachers in higher education use a variety of resources to shape their curricula and courses. Opportunities afforded by Open Educational Resources (OER) get more and more attention. OER can be defined as ‘*learning, teaching and research materials in any format and medium that reside in the public domain or are under copyright that have been released under an open license, that permit no-cost access, reuse, re-purpose, adaptation, and redistribution by others’* (UNESCO, 2020,1 par. I, point 1). OER have the potential to improve teaching and learning in higher education. Teachers, for example, have access to a wide variety of resources allowing them to vary their pedagogical and didactical approaches (Clinton-Lisell, 2021). In addition, students do not have to buy commercial resources, which means students might have equally access to quality materials (e.g. Wiley et al., 2016). This in turn could lead to an increase in OER-enabled pedagogy in higher education resulting in affordable and accessible education of good quality (Stagg, 2014; Wiley & Hilton, 2018). These affordances of OER have resulted in a wide array of initiatives around the world, but unfortunately not all of them turn out to be sustainable; many OER initiatives cease to exist once the initial project funding stops (Orr et al., 2015).

To support and encourage sustainable OER practices at national, regional, and institutional levels, UNESCO, in its Recommendation on OER (UNESCO, 2020), formulated ‘nurturing creation of sustainability models for OER’ as one of the five Areas of Action. One aspect of this specific Action focuses on ‘*promoting and raising awareness of other value-added models using OER across institutions and countries where the focus is on participation, co-creation, generating value collectively, community partnerships, spurring innovation, and bringing people together for a common cause’* (par. iv, point c)*.* In accordance with this recommendation, interest has increased in community building in relation to OER.

Communities on OER might have the potential to foster sustainable practices (Baas et al., 2019; Orr et al., 2015; Wang & Wang, 2017). Yet, such communities can only function if their members perceive these as valuable. If teachers do not feel that participation in a community gives them some value, engagement will decrease and the community will fall apart (Wenger et al., 2002). Hence, value creation is essential, as it can inform communities on cultivating and maximizing their value for participants (Wenger et al., 2011). With this in mind, the present study was set out to examine the value teachers ascribe to their participation in an inter-institutional community on OER and other related aspects of teaching. Our aim is to contribute to the understanding of cultivating value in order to make sustainable OER initiatives more common.

## Theoretical framework

### Towards sustainable OER communities

Sustainability of OER initiatives is a concern that has received considerable attention in recent decades. About 15 years ago, Downes (2007) specified that sustainability models relate to (a) the funding of the initiative, (b) the technical sustainability of OER related to the development and distribution of quality OER, (c) the content and the type of OER that impacts its lifespan, and (d) the selection and hiring of staff which is needed to cultivate and sustain the initiative. Recently, new insights on the evolution of sustainability models for OER in higher education have been presented by Tlili et al. (2020). They outlined 10 models, such as models that aim at gaining funding (e.g. internal or public funding), models that aim at generating funding (e.g. offering learning-related data to companies or producing OER on demand), and models that focus on communities (e.g. participation in an OER network). Although the authors clearly distinguish between the 10 models, they nevertheless stress that in practice institutes often implement a combination of some of these.

Regardless of these 10 sustainability models, the aspect of community building is paramount for all OER initiatives, as there must be a shared belief in the value of the collaboration (De Langen, 2018). Value can generally be described as importance, worth, or usefulness (Wenger-Trayner et al., 2019). Value creation is crucial as it determines whether teachers will engage with the OER initiative. This in turn will decide whether a community will grow and develop. Measures of success relate to the size of and the activities in a community, but these aspects of increasing the size of the user group and nurturing the creation of a community are the key challenges for OER initiatives (Orr et al., 2015).

These challenges are explored by previous studies that have examined enablers of community engagement. For example, Wang and Wang (2017) and Stagg and Partridge (2019) examined a community-based approach to foster the adoption of open textbooks into the curriculum. Their findings indicated that a deliberate strategy is needed with a dual focus on a supportive learning space for teachers to have discussions, generate ideas and to experiment with open textbooks, and on the role of facilitators to organize structured meetings and to connect teachers’ needs with information and expertise within the institute. In line with this, Baas et al. (2022a) showed the importance of brokers in cultivating an inter-institutional community on OER. Due to their personal, small-scale, and content-oriented approaches, brokers were pivotal in encouraging teachers to engage with the community.

Collaboration between universities can enable transformational change in higher education through which collaborative learning practices can evolve and social inequalities can diminish (Laufer et al., 2021). However, although some communities on OER flourish (e.g. MERLOT,^2^ CCCOER^3^), most of them simply tamp out. To foster the number of sustainable OER initiatives, we must strengthen our understanding of cultivation of communities on OER with specific and empirical insights into teachers’ perceived value.

### Value creation in communities

As mentioned above, communities are only viable for as long as their members experience value. For the viability of communities, value creation is essential: participation costs time, meaning that ‘*most community members experience both internal and external pressure to discover and deliver value soon after the community starts’ *(Wenger et al., 2002, p. 84). It is therefore important that organizations support the community by creating an environment in which participation is encouraged (Wenger et al., 2002). In this study, we explore a community on OER that has the structure of a community of practice in which teachers voluntarily collaborate and share knowledge and resources on a common topic. The community members are pivotal in maintaining continuous interaction and engagement and thereby determining the sustainability of the community, which means this collaboration should be perceived as valuable by the participating teachers.

**Fig. 1:**
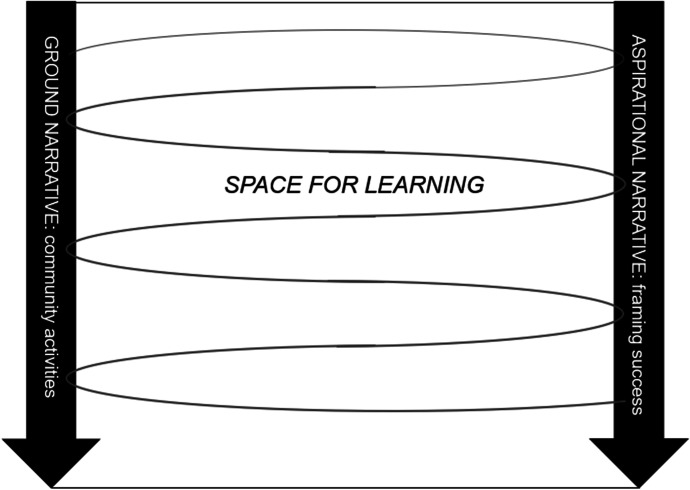
Productive tension between ground and aspirational narratives (based upon Wenger et al., 2011)

The value creation framework (Wenger et al., 2011) provides a structure to examine value creation in communities. This framework can be used as an analytical tool to examine in what ways teachers find value through their participation with a community. Personal and collective narratives can be collected to create an account of value creation. Two functions of these narratives must be considered. The ground narratives are stories of the members about the past and current everyday life of a community that has shaped the development of the community. For example, it includes the interactions that teachers have with others, and the activities they are involved in. The aspirational narratives are stories about what the community is expected to produce, which evolves over time. For example, it includes teachers’ individual expectations of what their engagement in the community will provide for them, as well as the collective value a successful community should provide. The tension between these ground and aspirational narratives creates a space for learning (see Fig. 1).

It is within this space that the following five cycles of value creation can be defined (Wenger et al., 2011): immediate value, potential value, applied value, realized value and reframing value. Table 1 describes these values in more detail.

**Table 1:** Value Creation Framework (Wenger et al., 2011)

Cycle	Value	Definition	Description
1	Immediate value	Community activities and interactions	Answer to a question, a solution to a problem, or help with a challenge
2	Potential value	Knowledge capital	
	a) Personal assets	Human capital	Useful skills, a key piece of information, or a new perspective. Personal value can be inspiration, caring, confidence, and status
	b) Relationships and connections	Social capital	Knowledge as a collective good distributed across the community. Social resources can facilitate learning and communication which can lead to opportunities for collaboration and the ability to promote a cause
	c) Resources	Tangible capital	Access to resources (e.g. documents, tools, procedures, links, etc.)
	d) Collective intangible assets	Reputational capital	Reputation of community, status of profession, collective voice or recognition it provides within an organization. These assets increase the potential for collective action
	e) Transformed learning	Learning capital	Valuable way of learning besides formal learning, transfer learning to other contexts
3	Applied value	Changes in practice	Adapting and applying knowledge capital. This can lead to changes in actions, practice, tools, approaches, or organizational systems
4	Realized value	Performance improvement	Performance improvement. Changes in practice does not guarantee performance improvement. Reflect on effects of application of knowledge capital
5	Reframing value	Redefining success	Redefining success and learning imperatives (e.g. reframing strategies, goals, values). Success can be redefined at individual, collective and organizational levels

These five value cycles are not hierarchical or mutually exclusive. The collaboration and interaction among teachers in communities can lead to perceived value in one cycle or in multiple cycles, and this does not imply that one value cycle is inferior to another.

These cycles of value creation have been explored in various studies on networks and communities and from several perspectives. Previous studies have, for example, examined value creation from the perspective of teachers (Booth & Kellogg, 2015; Van Waes et al., 2016; Zaalouk et al., 2021), students (Dingyloudi et al., 2019; Forbes, 2020; Mavri et al., 2021), volunteers (Hanley et al., 2018), and participants in a cross-border learning network (Clarke et al., 2021). However, within the domain of OER, insights into value creation within communities are scarce. Although earlier studies have focused on communities on OER (Borthwick & Dickens, 2013; Burgos-Aguilar & Mortera-Gutierrez, 2013; Smith & Lee, 2017; Tosato & Bodi, 2011; Tosato et al., 2014), these studies merely revolved around initiating and realizing the community. Little insights are available that explored the value teachers ascertained to their engagement within such communities. Yet, improving our understanding on the question that teachers might ask themselves: ‘what’s in it for me?’, would be beneficial to foster sustainable OER communities.

## Method

The purpose of the present mixed-methods study was to characterize the value creation that occurred within the inter-institutional community. The findings of this study will provide insights into the different value cycles that can be provided by communities on OER, which may help to expand our understanding of the sustainability issues of OER initiatives.

### Research context

Since 2015, policies in the Netherlands have focused on supporting OER in higher education (OCW, 2019)^4^. Subsequently, the Dutch government initiated a national funding program by which higher education institutes were encouraged to explore inter-institutional collaboration on OER. In this mixed-method study, we explored one of these projects in which 15 universities of applied sciences collaborated on sharing knowledge and resources across their institutions within the domain of Nursing Education. The aspirational narrative of this community was to realize a sustainable OER initiative in which sharing and reuse of OER within an active professional community of teachers across institutes is common practice.

**Fig. 2:**
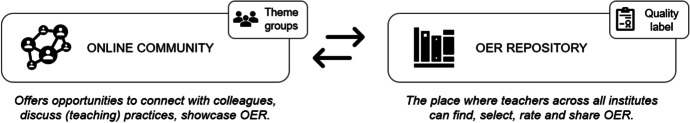
Context of the study

As collaboration within communities on OER does not happen spontaneously (Tosato et al., 2014), two interconnected digital platforms were used to promote and support engagement and interaction: an online community and an OER repository (see Fig. 2). In the online community, teachers could connect with colleagues, discuss OER and teaching practices, or articulate needs for collaboration. Various thematical groups were created in which teachers could connect and discuss certain themes. In the OER repository, teachers could search and share resources; a quality label was provided for resources that met predefined quality criteria. In addition to these technological platforms, each institute allotted brokers as a linking pin between the project and the institutes to cultivate the community.

This community originated upon existing Nursing Education networks. By utilizing these existing networks, the sustainability of the initiative could be more feasible (Schreurs et al., 2014). Sustainability was also pursued through institutional funding after the initial national funding (2018–2020) had ended.

### Research design

**Fig. 3:**
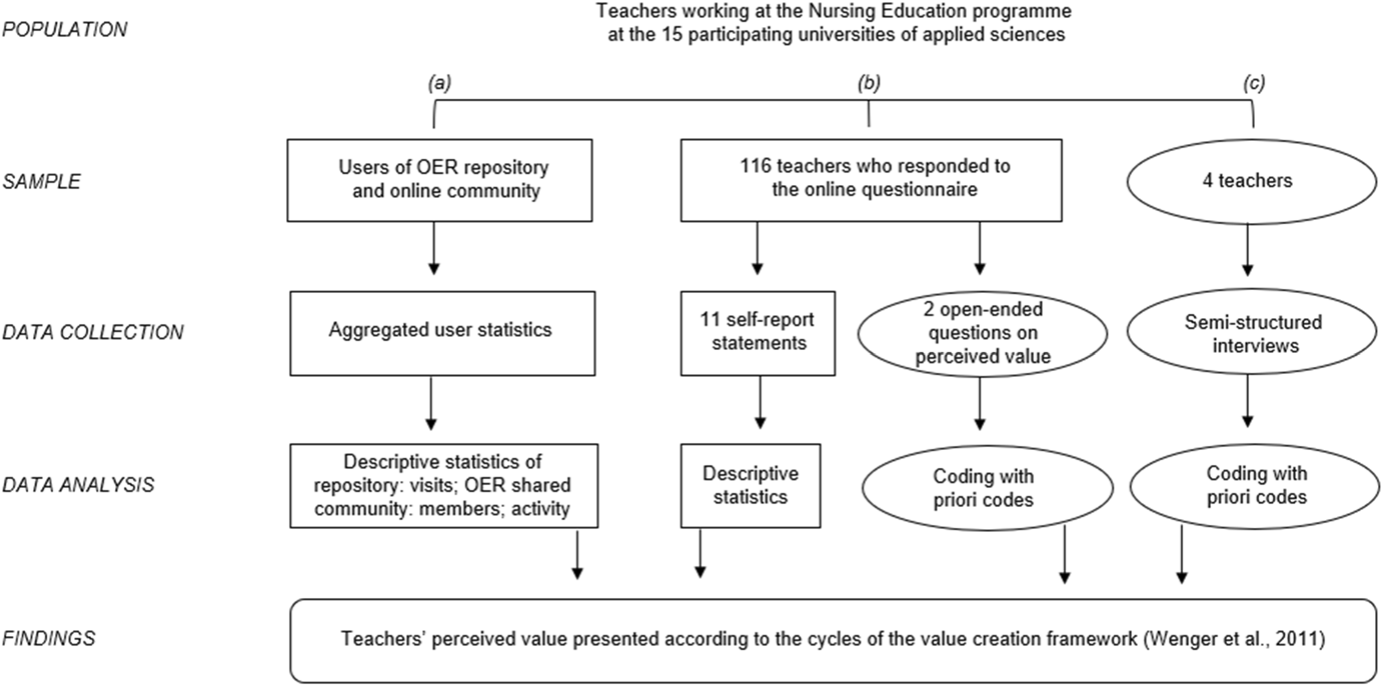
Visualization of the data collected in this mixed-method study

We applied a convergent design (Creswell & Clark, 2018) in which both qualitative and quantitative data were collected in the same time period. The value creation framework allows the inclusion of various types of data (Wenger et al., 2011). In this study, data were gathered by (a) downloading user statistics of the OER repository and the online community, via (b) an online questionnaire, and through (c) semi-structured interviews with teachers, the users of the platforms. A visualization of the mixed-method design is provided in Fig. 3.

### Procedure and participants

Ethical approval was obtained from ICLON-Graduate School of Teaching at Leiden University before conducting the study. Teachers were recruited from all 15 universities by open calls distributed within the online community and through the installed brokers within the institutes. An additional call to participate in the interviews was included at the end of the online questionnaire. The questionnaire was open for all teachers to participate in. For the interviews purposive sampling was employed: Teachers who participated in the OER community, and were not part of the project organization were eligible.

The questionnaire was available for teachers late September to mid October 2020. Participation was voluntary and data collection was anonymous as teachers were invited indirectly. The questionnaire had a (partial) response of 116 teachers. Among them, the majority were female (87.9%, n = 102), which is representative with respect to the demographic statistics of nurses in the Netherlands (CBS, 2019)^5^. Table 2 provides the general characteristics of the participants.

**Table 2:** General characteristics of participants in questionnaire (N = 116)

Characteristics	Categories	Total (n/%)
Gender	Male	14 (12.1)
	Female	102 (87.9)
	Other	0 (0.0)
Age	< 26 years	1 (0.9)
	26 – 35 years	34 (29.3)
	36 – 45 years	27 (23.3)
	46 – 55 years	29 (25.0)
	> 55 years	25 (21.6)
Teaching experience	0 – 2 years	27 (23.3)
	3 – 5 years	36 (31.0)
	6 – 10 years	28 (24.1)
	> 10 years	25 (21.6)

For the interviews, a small sample was chosen because of the expected difficulty in obtaining teachers willing to participate due to the Covid-19-pandemic. Most teachers were either helping out in healthcare organizations or were fully occupied with the switch to online education. A total of seven teachers responded to the calls, but two teachers had to withdraw and one teacher was closely involved in the project organization and did therefore not meet the sampling criteria. In the end, four teachers participated in the interviews. Table 3 present the fictional names and background characteristics of these teachers.

**Table 3:** Demographics and pseudonyms of the participating teachers in the interviews

Teacher	Age	Teaching experience
Marisa	53 years	17 years
Simone	48 years	17 years
Dafne	44 years	18 years
Will	57 years	15 years

When inviting the participants, the purpose of the research was clearly explained. On obtaining informed consents from the teachers, an online meeting was scheduled. The interviews lasted between 30 to 40 min. The interviews were summarized and sent for member checking purpose. One teacher made minor changes, which related to the type of resources found in the repository.

### Data collection

Through user statistics (3.4.1), a questionnaire (3.4.2), and interviews (3.4.3) we collected data in relation to the five value cycles. An overview of the different data sources for each value cycle is presented in paragraph 3.4.4.

#### User statistics

We collected user statistics to gain insights on teachers’ participation in the platforms. Data of two indicators relating to immediate value were collected: level of participation, and level of activity (Wenger et al., 2011). For the OER repository, we had access to the number of page visits, and the number of OER shared. For the online community, we gathered the statistics on the number of members, and teachers’ online activities. Only aggregated data were collected; no personal data were accessed.

#### Questionnaire

The questionnaire was designed to ascertain teachers’ value creation in the community on OER. See the data availability statement for a link to the questionnaire and data collected. We included several pre-structured self-report questions and statements to assess teachers’ engagement and value creation. We included items that were developed based on the OER Adoption Pyramid (Cox & Trotter, 2017) as no quantitative measurement tool exists to measure value creation. We related these OER specific items to several value cycles (see Table 4).

Two open-ended questions were included to collect teachers’ perceived value of both the OER repository and the online community. If teachers had used the repository, they were asked to describe the value of it for their practice and to give an example how it had affected their work. The same questions were posed if teachers had used the online community.

The questions and the order thereof were discussed with the project manager of the inter-institutional community to ensure face validity. Afterwards, the items were discussed in the research team to ascertain content validity.

#### Interviews

The semi-structured interviews were conducted to gain detailed insights into the perceived value of the community on OER. We used the value creation framework (Wenger et al., 2011) to design the interview guide. The guide consisted of questions that were intended to collect teachers’ value creation stories of both the repository and the online community. We asked generic starting questions that permitted the teachers to tell us their experience (e.g. can you tell me how you have used the repository?; how did the online community influence your practice?) after which the interviewer asked for elaboration or explanation when needed. At the end of the interview, teachers had the opportunity to express any additional thoughts. All interviews were conducted by the first two authors.

#### Overview

Table 4 presents the main data sources for each of the five value cycles. In addition, overarching questions were asked in both the questionnaire (e.g. can you give an example how online community has influenced your practice? What have you gained from it?), and the interviews (e.g. can you give an example of how this influenced your practice?).

**Table 4:** Overview of main data collected within value cycles

Cycle	Data source	Data collected
Immediate value	User statistics	Repository: number of page visits; number of OER shared
		Online community: number of members; online activities
	Questionnaire: self-report statements	How would you characterize your usage of the repository?
		How would you characterize your usage of the online community?
		What activities have you undertaken in the online community?
		I have shared resources in [the OER repository] or arranged for them to be shared (e.g. by the library)
	Interviews	Can you describe how you use the online community?
		Can you describe how you use the OER repository?
Potential value	Questionnaire: self-report statements	I know how to search for resources in [the repository]
		In [the repository] I can find resources that are relevant
		In [the repository] I can find resources of good quality
		I plan to (continue to) use resources from [the repository] in the future
		I know the conditions under which I may reuse resources from [the repository] in my own teaching
	Interviews	Through teachers stories in which prompts were used to prodding further storytelling
Applied value	Questionnaire: self-report statements	I have used resources of [the repository] in my own education without making adjustments to them
		I have used resources of [the repository] in my own education with making adjustments to them
		I use the quality mark to determine the quality of resources in [the repository]
	Interviews	Through teachers stories in which prompts were used to prodding further storytelling
Realized value	Interviews	Through teachers stories in which prompts were used to prodding further storytelling
Reframing value	Interviews	Through teachers stories in which prompts were used to prodding further storytelling

### Data-analysis

For the quantitative data, descriptive analyses were carried out on the user statistics data and the answers on the pre-structured questions of the questionnaire. Data from the open-ended questions in the questionnaire and the interview were analysed in Atlas.ti through two coding cycles (Saldaña, 2016). First, to gain sense of the data, we explored the transcribed interviews through a combination of process coding and evaluation coding. This enabled us to gain a first general impression about both the actions of teachers within the inter-institutional community and their judgments about the (non)merit and (non)worth of it. For the second cycle of coding, we developed a coding scheme based on the conceptual framework on value creation (Wenger et al., 2011). In several iterations, fragments within both the open-ended questions and the four interviews, were selected with these priori codes. Between iterations, the initial coding was discussed in the research team to gain consensus on the labelling of the selected fragments. The main disagreements in coding resulted from differences in interpretation of the codes ‘immediate value’ and ‘realized value’. After modifying the labels that we allocated to the value cycles codes, the data were again analysed which resulted in a total of 145 labelled fragments. Some fragments received simultaneous coding in which multiple codes were assigned to parts of the transcribed text due to an overlap of multiple value cycles. This is in accordance with previous studies in which segments of narratives were not always exclusive for one value creation cycle (Booth & Kellogg, 2015; Mavri et al., 2021). This led to descriptions of the value creation within the five value cycles. Lastly, we revisited the data to visualize the value creation across cycles based upon the narratives of the interviewed teachers. We defined the relationships and continuity of their ascertained values based upon their storytelling. This enabled us to illustrate how a teacher’s value creation traversed cycles.

## Findings

The main findings are structured based upon the five value creation cycles. Prior to these, we present the interviewees’ value creation stories to illustrate how value creation can occur across cycles (4.1). Then, we present our main findings, which include all data following the five cycles of value creation (4.2 – 4.6). Where applicable, each of these sections begins with the presentation of the quantitative data. Detailed insights are provided for each value cycle based on the qualitative data.

### Value creation stories

**Fig. 4:**
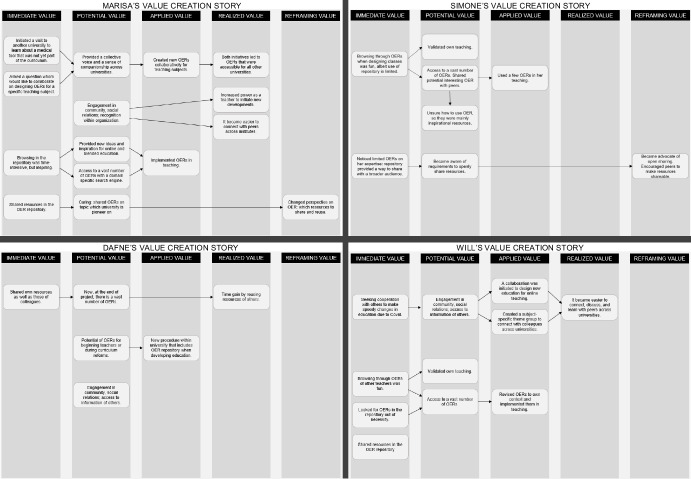
Value creation stories of the interviewed teachers

To better understand how the community is creating value, we examined the personal narratives of Marisa, Simone, Dafne, and Will and visualized their stories in Fig. 4.

It became clear that each teacher ascertained different types of values to their participation in the inter-institutional community, but that there are also similarities across teachers. For example, Marisa, Simone, and Will mentioned that browsing the repository was fun and inspiring (immediate value). It provided them with access to a vast number of resources which resulted in either inspiration or a means to validate their teaching (potential value), whereas other OERs were implemented in their actual teaching practice (applied value). With regards to sharing resources, the four teachers mentioned that they had shared their resources in the OER repository. It primarily provided them with an immediate value of being able to share their resources to a wider community rather than only with their own students or colleagues. For Simone this also led to an awareness of the requirements of sharing OER (potential value), and she explained that within her team she became an advocate of OER (reframing value). For Marisa sharing resources also led to a redefinition of success. She shared her resources on a topic in which the university is a pioneer so that others could use and learn from them (potential value). At the same time, this also resulted in a personal redefinition of what Marisa believed are quality resources (reframing value). A final similarity is related to the potential value of social relationships as revealed in the stories of Marisa, Will, and Dafne. Will and Marisa explicitly mentioned that the OER community led to improvement of their practices, because connecting with peers across institutes for queries or collaboration became easier (realized value).

It is apparent from this figure that most value was created across the immediate, potential, and applied value cycles, whereas less value was created in the realized and reframing values. These value creation stories are useful to understand how value can traverse cycles from an individual perspective. Yet, a more complete picture of the value created by the community can be obtained by combining data for each value cycle. Hence, in the next sections we present the value cycles from a collective narrative by inferring from all data.

### Cycle: Immediate value

Immediate value is about ‘*networking/community activities and interactions as having value in and of themselves*’ (Wenger et al., p. 19). We first present the findings based on the quantitative data (4.2.1), after which we elaborate on the qualitative findings (4.2.2).

#### Quantitative data

**Fig. 5:**
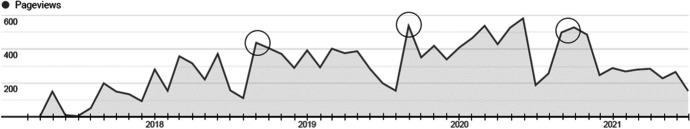
Pageviews of the homepage of the OER repository (circles added)

An indication of immediate value of the repository can be derived from the user statistics. The pageviews of the OER repository homepage, for example, show an increase of online traffic, despite the high and lows, between 2018 and mid 2021 (see Fig. 5). Traffic was relatively the highest at the start of each academic year (see added circles), and during the lockdown period in Spring 2020. After the end of the project in November 2020, pageviews appeared to have declined and stabilized.

In the questionnaire, teachers (n = 65) characterize their usage of the repository mostly as very occasional (47.7%) or occasional (23.1%). Teachers can share and search for resources. In July 2021, a total of 1458 resources were shared in the repository, including third parties resources.

**Fig. 6:**
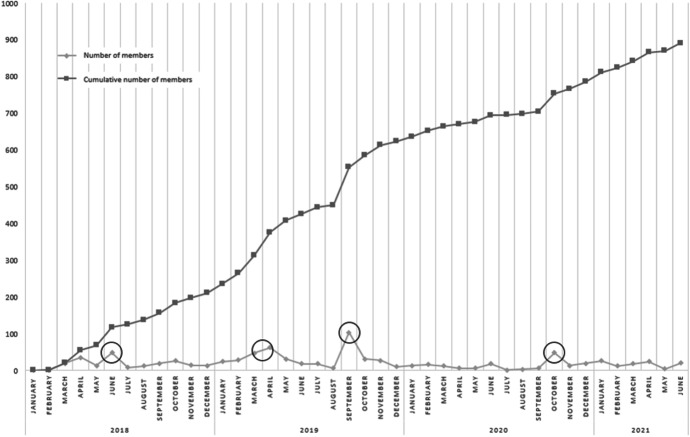
Growth of members of the online community

In addition of the value of the repository, an indication of the immediate value can also be derived from the user statistics of the online community. Since the start of the project in 2018, the number of community members gradually raised to a total of 891 users in July 2021 (see Fig. 6). The data show (see added circles) that the month after the start of the project (June 2018), Spring 2019, and the start of the academic years in 2019 and 2020 had the highest increase of new members. The number of new members continued to increase after the end of the project in November 2020.

Teachers characterize their use of the online community (n = 64) in the questionnaire as very occasional (51.6%) and occasional (26.6%). With respect to activities undertaken in the online community, about half of the teachers stated that they had joined a theme group (n = 30) or looked for specific information (n = 20), whereas about a third indicated that they had created (n = 19) or responded to a post (n = 19).

**Fig. 7:**
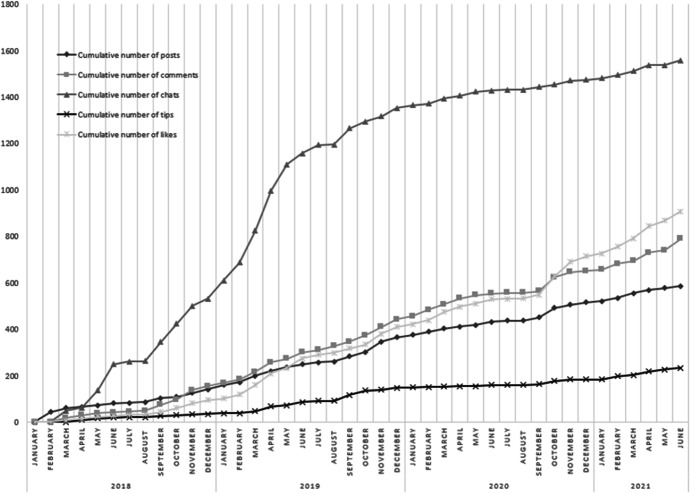
Activity within the online community between January 2018 and July 2021

If we investigate the user statistics of the online community it is apparent that activity gradually increased between January 2018 and July 2021 (see Fig. 7), in line with the increase of new members. In total, online community members created 586 posts and received 789 comments and 907 likes. Teachers could also send a person or a group a so-called tip to draw someone’s attention to a post, which was done 234 times. The highest number of activities relate to the chat messages: the online community groups sent 1557 chat messages. Interaction within the online community continued after the official project ending late 2020.

#### Qualitative data

A common view amongst teachers was that the repository provided them with a welcome opportunity to browse through resources of peers, as became visible in their answers on the open response question relating to the repository (n = 31) and in the interviews (n = 4). Some teachers also stated that the exploration of these resources led to an ancillary value of validation of their teaching approaches. For example, Simone explained that: *‘[…] you see a lot of familiar things and you think, yes, that doesn't contribute anything new, that's how we do it too. So it can also be valuable to be acknowledged for that what you do, you do well.’* Another value came up in the interview with Will who explained that the repository also served to face a sudden unexpected challenge when *‘I suddenly had to take over a class of a colleague, […] and then I had to familiarise myself [with the content] and think of resources that I could use in my teaching’.* With regards to sharing resources, a variety of perspectives were expressed. Teachers valued that they could share their resources with a broader audience than just their own students; increase the number of resources on topics that were underrepresented in the repository; and showcase their resources.

Nevertheless, despite the perceived value of the repository, two concerns were expressed regarding searching OER in the repository. First, several teachers felt that searching and sharing OER was time sensitive and difficult, because finding *the* OER proved to be challenging. Second, there were some negative comments about the quality of the resources as it was experienced that numerous resources were either too context specific (e.g. includes school-specific information) or too narrow (e.g. one small assignment without instructions).

With regards to the online community, teachers’ answers on the open-ended question relating to the value of the online community (n = 18) and the interviews (n = 4), showed the value of connecting with peers across institutes. Many of the teachers indicated that they used the online community to ask questions, to receive tips, to connect, or to get help with a challenge. For example, in the interview with Dafne, she emphasized this value by explaining that a colleague of her: *‘really appreciated that he had a network of people. […] At our institute, there are only three of them I believe, so then it is really great to see what others are doing.’* Marisa mentioned that she used the online community to initiate a school visit to learn more about an educational tool: *‘I knew about an institute that had created a [tool] at the time. […] and then we had a chat about that and when I planned to visit the institute, […] I said to the other institutes “I’m going there on Monday, do you want to come too?”’.*

### Cycle: Potential value

The OER community can also produce value that is not immediately realized when the value *‘lies in its potential to be realized’* (Wenger et al., p.19). Potential value can be distinguished in five subcategories: tangible capital, human capital, social capital, reputational capital and learning capital. Findings on each of these categories are presented except for learning capital, because we did not identify this in the data.

#### Tangible capital

In the questionnaire, several statements related to tangible capital, which is the access to resources. More than half of the participants (60.0%) agreed with the statements that they know how to search for resources. A small number of teachers indicated that they cannot find resources that are relevant (18.4%) or of good quality (10.8%). These concerns were also expressed in Section 4.2.2.

However, the qualitative data showed that the potential value of this access to resources is significant. In the open-ended question relating to the repository (n = 31) and the interviews (n = 4), almost all teachers mentioned that it provided them with an excellent way to access other’s resources. It was suggested that this value increased due to the Covid-19-pandemic, because it required teachers to transfer to online teaching and blended learning. For example, Marisa explained that students miss their peers and *‘now we are thinking […] to work with learning communities to foster the group cohesion in a different way. Then you have to come up with a lot more small assignments and then I see that there are resources available in [the OER repository]’.* A small number of the teachers also signalled the potential value of the repository for future curriculum reforms. Dafne for example, explained that: *‘next year a curriculum reform is on the agenda, so I think we will definitely make colleagues enthusiastic about [the OER repository] […].’*

#### Human capital

Yet, a lack of knowledge about what is allowed was an impediment for OER adoption. The data of the questionnaire (n = 65) showed that about a third of the teachers (35.4%) did not know under which conditions they may reuse resources.

Indeed, this is underlined by the qualitative data, as several teachers mentioned that they would have liked to reuse resources, but did not know what was allowed. For example, Simone said: ‘a*nd that’s why I didn’t use [the resource] as-is, because I didn’t know exactly what was allowed. I did use it as inspiration though. That also feels a bit weird, because […] you are using someone else’s resource, but I don’t explicate that anywhere.’* Overall, teachers argued that the main personal value was the inspiration that these resources gave them. Teachers learned new educational tools, got ideas of ways to present their teaching content, gained insights what other institutes were teaching, or learned about other pedagogical approaches. Some teachers took up new perspectives about education as they alluded to the notion of online learning as a sustainable component of future education. They experienced that this inter-institutional collaboration enabled them to choose from a plethora of resources that can support this transition to online learning. A few teachers reported that the access to resources made them feeling competent. Commenting on this, one teacher in the questionnaire explained: *‘the realisation that I’m not doing so badly after all. I still find it “scary” to share something. My own insecurities. I will stop that.’*

#### Social capital

The potential for relationships and connections is considerable, because from the qualitative data (open-ended question relating to the online community (n = 18) and the interviews (n = 4)), it became clear that all teachers acknowledged the potential to connect with peers from other institutes as a major asset. Their view was that sharing developments and issues and connecting with teachers within the same subjects across institutes is invaluable. For example, Dafne mentioned: *‘I would say that one plus one is three. That if you share, you end up with more. That is also why I am enthusiastic about it; two people know more than one.’* A small number of teachers stressed that the current community is not yet mature enough. As one teacher in the questionnaire stated: *‘it has the potential to be a great asset as it makes it easy to connect with colleagues that focus on the same subject and to learn from each other. Though, it is not yet used enough and is it too quiet to be a proper community.’*

#### Reputational capital

In the interviews, Marisa reported that the inter-institutional community provided her with a potential of a collective voice for action. She, for example, emphasised that it offered a voice to put forward the development of much-needed resources because: *‘it is absurd that I work with a [tool] in the hospital and [it is used] in every health care organization, but that we don’t have it in education. So, this was such a pressing matter that we thought, that has to be implemented straight away.’*

### Cycle: Applied value

This value cycle focuses on *‘adapting and applying knowledge capital in different contexts [that] can lead to changes or innovations in actions, practice, tools, approaches, or organizational systems’* (Wenger et al., p.20).

One of the expected changes in practice relate to the use of OER. Indeed, the quantitative data showed that reuse had occurred, albeit limited. From teachers’ response on statements relating to reuse of OER (n = 63), only a few teachers used resources, either with (15.9%) and without (6.3%) adjusting them. Another statement related to the use of the quality mark that was provided as a tool to advice teachers about high-quality resources. However, of the users of the repository (n = 65), only a small number of teachers had used this mark when searching for OER (15.4%).

Nonetheless, the open-ended question answers (n = 31) illustrated that the teachers who adopted OER were positive about its impact. One teacher reported that it provided: *‘great assignments, resources and tips […]. I regularly use parts of existing resources and revise them where necessary for my own lesson. [The OER repository] is of value for new lessons where our school does not yet have any resources available.’* This was echoed by the teachers who were interviewed as Marissa, Simone and Will all have adopted OER in their teaching. And although Dafne did not make any changes in her practice, she explained that some organizational structures within her institute were changed to foster OER adoption. She made clear that *‘the curriculum committee has a procedure for the development of new education which states that [teachers] should first look in [the OER repository] before we start to develop.’*

Another recurrent theme in the interviews was a sense of collegiality. They mentioned an increased awareness of the fact that colleagues might encounter the same issues or have similar desires. Talking about this, Will explained that students were not able to travel abroad due to Covid-19, so an alternative program had to be designed on a relatively short notice. He used the online community to connect across institutes and now teachers from several institutes are *‘exploring whether we can create an alternative program for students […]. And then it is nice to be with a group of people that share the same professional background and who can think along in potential good assignments.’*

### Cycle: Realized value

In the previous section, we presented teachers’ changes of their practice, but these changes do not necessarily imply improvements. Realized value explores *‘what effects the application of knowledge capital is having on the achievement of what matters to stakeholders’* (Wenger et al., p.21). Two themes related to improvements were identified in the open response questions and the interviews. First, teachers mentioned that it became easier to approach and connect with teachers from other institutes to ask questions or to share and discuss issues. For example, Marisa explained that the community *‘is a very direct way of talking to people and meeting them. And that others say “I have heard this and that, or that institute is also working on it” and before you know it, you have another email address that you have access to.’*

The second aspect of improvement relate to the increased power that the inter-institutional OER community has provided teachers within their institutes. Marisa reported that it offered her a platform to initiate a new collaboration between various institutes to create OER for skills that are vital in students’ future profession, but that are not a part of the curriculum. She explained this increased power by saying: *‘it is often the case that things are developed from a theoretical point of view, but then it is debatable whether it has any real added value in the primary process [of teaching]. Whereas now, I notice that the gap between theory and practice closes somewhat because the needs are positioned lower in the primary process.’*

### Cycle: Reframing value

Reframing value refers to *‘a reconsideration of the learning imperatives and the criteria by which success is defined’* (Wenger et al., p. 21) which can occur on individual, collective, and organizational levels. In the interviews, we identified two reframing values, both on a personal level. The first example is Marisa who redefined her perception of reusable resources. She clarified that when they were encouraged to upload OER, she started to think about *‘what are good resources to share? And only then did I get a more critical view of what I do and do not use’*. The second example is Simone who became an advocate for open sharing. As she became more acquainted with the requirements of open sharing, she pro-actively approached colleagues to point out what should be improved so that the resource could be shared in the repository.

## Discussion and conclusion

This convergent mixed-methods study was set out with the aim of providing insights into value creation in an inter-institutional community on OER in higher education. Previous studies have examined the initiation and the realization of such communities, but our understanding of the value that teachers ascertain to their engagement with communities on OER is limited. Yet, the insights thereof may help to expand our knowledge of increasing value creation in OER communities so that teachers continue to engage with them. Hence, we applied the value creation framework of Wenger et al. (2011) to illuminate ‘the added value *for* community members as defined *by* community members’ (Dingyloudi et al., 2019, p. 217).

### Teachers’ perceived value: What’s in it for me

The findings of our study illuminate that value, traversing all five value cycles, was created in the OER community. By combining data, an account of teachers’ experienced value creation could be formulated. A main finding to emerge from the analysis is that major value creation occurred from teachers’ personal needs, resulting in dominant immediate and potential values. Teachers experienced value because their participation in the inter-institutional community resulted in access to resources, inspiration, connections with peers, or aid during emergency teaching. The repository provided teachers with access to relevant resources that they could use in their own teaching, either when designing a lesson, for some last-minute changes, or during curriculum reforms. Teachers especially mentioned the value during the school closures during the Covid-19-pandemic, which might be obvious because teachers had to suddenly switch to online education. OER communities, therefore, not only provide value and support in teachers’ day-to-day practices, but also in crisis situations (see also Zaalouk et al., 2021).

In this study, two loosely coupled platforms operated as the foundation of the OER community: teachers could find and share resources in the *repository*, and they could connect, ask questions, or discuss practices with peers in the *online community*. We underline the necessity of collaboration-supporting technology because it transcends space and time to connect institutes across their physical borders, but it also enables institutes to include elements of work practices (e.g. standards, cultures) into school practices (Mavri et al., 2021). The latter is especially relevant for some programmes of higher education institutes because more emphasis is placed upon creating authentic learning environments at the school-work boundary to better prepare students for occupational practice (Bos, 2022; Bouw et al., 2021).

OER communities might facilitate boundary crossing across institutes (Baas et al., 2022a). Findings on applied and realized value denote that it became easier for teachers to connect with peers, and to initiate collaboration projects across institutes, because boundaries between institutes had diminished. Indeed, all four boundary spanning mechanisms that foster the connectedness between institutes (Hawkins & Rezazade, 2012) were employed within the context of this study: boundary objects (e.g. OER), boundary spanning (e.g. brokers), boundary discourse (e.g. teachers’ conversations in the online community), and boundary practice (e.g. initiation of collaboration across institutes). Yet, Hawkins and Rezazade (2012) emphasize that these spanning mechanisms evolve over time. This could explain why less realized and reframing values were identified in our data, in line with previous studies (Booth & Kellogg, 2015; Forbes, 2020; Van Waes et al., 2016). In our case, it could be that it was too early to discern these values because teachers were still getting acquainted with the community, which might take longer to transpire. It could also be that teachers do not yet articulate these values, as it requires them to reflect upon abstract notions and phenomena of success (Dingyloudi et al., 2019).

### Value creation to inform sustainable practices of inter-institutional communities

We present three practical recommendations that could support communities on OER to cultivate sustainable practices. These recommendations relate to the sustainability of (1) the community’s aspirations, (2) the connection of it with the wider organization(s), and (3) OER adoption.

First, the value creation as defined by its members can not only be an inspiration for its members but can also inform community managers and higher education institutes to further sustain the community by designing supporting activities and practices (Wenger et al., 2011). For example, in our study most value was created in the immediate and potential value cycles. Although values are not mutually exclusive, changes in their practice remained constrained compared to the aspirational narrative of the community (see 3.1). We recommend that communities use the framework to look forward and examine how further value creation can be promoted. For example, within this context, community leaders can decide to commence actions that encourage teachers to experiment with OER in their teaching practices. To stimulate such a change, it is important to create an awareness of the broader change process including the transition from traditional teaching practices to open teaching practices (Schophuizen & Kalz, 2020).

Second, in line with the first recommendation, it is vital that the value creation of the community is in line with the developments within the wider organization(s). If there is no alignment with burning issues of the organization(s), the community will still have value for its members, but there will be no or limited managerial support (Büchel & Raub, 2002). Büchel and Raub argue that without management support, sustainable practices in which members learn from each other simply cannot evolve. A key priority for communities should, therefore, be to connect and align its narrative with the wider organizational structures, visions, and issues, thereby aiming to extend the initial lifespan of the project. It could be helpful to repeatably and frequently assess value creation, and to use this information to further cultivate the community (Wenger-Trayner et al., 2019).

Third, we suggest that OER communities include teachers’ expectations and demands regarding OER when cultivating the community. Many OERs were shared within the context of this study, however, reuse remained limited. Despite the development of a quality model, it appeared that quality remained an issue for some teachers: OERs were perceived as not suitable as they were either too context-specific or too small. This relates to the juxtaposition of reusable resources; better known as the reusability paradox (Frantiska, 2016; Wiley, 2002). The reusability paradox describes that *‘if a [resource] is useful in a particular context, by definition it is not reusable in a different context. If a [resource] is reusable in many contexts, it is not particularly useful in any’* (Norman, 2003). This paradox means that if someone is designing an OER, they have to make the choice to either create an OER with little context in it that is easier to reuse but requires more of the users to personalize and contextualize; or to create an OER with much context in it, which better supports learning but also limits reuse. In the context of this study, a quality mark was developed to support teachers in designing OER as well as to find quality OER. Although this quality mark indicates a certain quality standard of a resource, the value of an OER still remains a personal assessment. To foster OER adoption, Baas et al. (2022b) suggest that conversations on OER in teacher teams might be a promising method. We recommend communities to organize such conversations, in which we stress the importance to include the support of librarians and instructional designers. Although OER in the current study were already context-specific (i.e. nursing education), they still need to be localized and personalized to align it with the teacher’s specific content and context (Hood, 2018). Especially support from instructional designers is needed because the pedagogical effectiveness of OER in practice does not only relate to the reusability of a resource, but also to the revisability of it to effectively support the student’s learning journey (Sandanayake, 2019; Wiley, 2020).

### Limitations and future research

This study has some limitations that must be addressed. First, we were able to interview four teachers who made use of the community, but all four of them were highly experienced teachers. Although we invited novice teachers as well, we failed in this due to the implications of Covid-19 pandemic on nursing education teachers’ teaching and healthcare obligations. Future research could therefore explore if and how value creation within communities on OER might differ for experienced and novice teachers, because experienced teachers have, as opposed to novice teachers, the means to actively shape their interactions to create realized and reframing value (Van Waes et al., 2016).

Second, future longitudinal research can deepen our understanding what is needed to mainstream OER. Our findings, based upon one single data collection moment, indicate that a community-approach might be a promising way to foster continuous engagement of teachers. This may lead to sustainable communities, but value creation must be actively nurtured throughout the evolution of the community. Longitudinal research could deepen our understanding how value creation changes over the life of a community (Wenger et al., 2002) and whether networks, collaborations, and alliances in higher education differ in this (Williams, 2017).

Third, we acknowledge that value creation might be different for other settings and other types of communities. This study was conducted within a specific context: teachers were voluntarily engaged in the inter-institutional community, and there were no set objectives, structured activities, process facilitators, or face-to-face moments through which we could relate teachers indicated value creation to certain activities or actions. The value creation framework yielded us with important understandings of the value that is created by the community on OER, but we also encountered some challenges, especially related to the allocation of value cycles to data fragments. We therefore agree with Booth and Kellog (2015, p. 695) that ‘*while the distinctions [between value cycles] can easily be understood conceptually, teasing out these distinctions within stories occasionally provide challenging.’*

## Concluding remark

This study emphasizes the importance of exploring value creation in an inter-institutional community on OER, and that the framework we used is helpful to inform actions to further promote value creation. Within this process, it is vital to connect the activities and connections that teachers deem valuable, the ‘what’s in it for me’, with the burning issues of the organisation(s) to promote sustainability.

## Data Availability

The questionnaire and the related quantitative data generated and analysed during the current study are available in the DANS repository, 10.17026/dans-xhg-nm73. The qualitative data supporting the findings of this article are not publicly available due to privacy reasons, but are available from the corresponding author on reasonable request.

## Footnotes

1 United Nations Educational, Scientific and Cultural Organization.

2 Multimedia Educational Resource for Learning and Online Teaching, an international community of educators, learners, and researchers.

3 Community College Consortium for OER, a community of practice for open education in North America.

4 Dutch Ministry of Education, Culture and Science.

5 Statistics Netherlands (CBS) is the national statistical office which provides reliable statistical information and data.
